# PAPupuncture has localized and long-lasting antinociceptive effects in mouse models of acute and chronic pain

**DOI:** 10.1186/1744-8069-8-28

**Published:** 2012-04-23

**Authors:** Julie K Hurt, Mark J Zylka

**Affiliations:** 1 Department of Cell and Molecular Physiology, UNC Neuroscience Center, The University of North Carolina at Chapel Hill, CB #7545, Chapel Hill, NC, 27599, USA; 2 The University of North Carolina at Chapel Hill, CB# 7545, 115 Mason Farm Road, Chapel Hill, NC, 27599-7545, USA

**Keywords:** Prostatic acid phosphatase, Adenosine A1 receptor, Acupuncture, Nociception, Ectonucleotidase, Popliteal fossa, Regional anesthesia, Phospholipase C

## Abstract

Acupuncture has been used for millennia to treat pain, although its efficacy and duration of action is limited. Acupuncture also has brief (1–2 h) antinociceptive effects in mice and these effects are dependent on localized adenosine A_1_ receptor (A_1_R) activation. Intriguingly, adenosine 5’-monophosphate (AMP) is basally elevated near acupuncture points. This finding suggested that it might be possible to inhibit nociception for a longer period of time by injecting prostatic acid phosphatase (PAP, ACPP) into acupuncture points. PAP is an ectonucleotidase that dephosphorylates extracellular AMP to adenosine, has a long half-life *in vivo* and is endogenously found in muscle tissue surrounding acupuncture points. Here, we found that injection of PAP into the popliteal fossa—a space behind the knee that encompasses the Weizhong acupuncture point—had dose- and A_1_R-dependent antinociceptive effects in mouse models of acute and chronic pain. These inhibitory effects lasted up to six days following a single injection, much longer than the hour-long inhibition provided by acupuncture. Antinociception could be transiently boosted with additional substrate (AMP) or transiently blocked with an A_1_R antagonist or an inhibitor of phospholipase C. This novel therapeutic approach—which we term “PAPupuncture”—locally inhibits pain for an extended period of time (100x acupuncture), exploits a molecular mechanism that is common to acupuncture, yet does not require acupuncture needle stimulation.

## Background

Acupuncture is a labor-intensive technique that entails insertion of fine gauge needles into specific locations of the body followed by intermittent manual rotation or electrical stimulation of the needles [1]. Essentially all acupuncture points are located in muscle and are in close proximity to peripheral nerves [2]. The axons of nociceptive (“pain-sensing”) neurons course through peripheral nerves [3-5]. This proximity of acupuncture points to nociceptive afferents could explain why acupuncture is modestly effective at treating pain in humans [1,6-8].

Recently, Goldman and colleagues found that the antinociceptive effects of acupuncture in mice were dependent on the localized activation of A_1_R [9]. Intriguingly, extracellular AMP concentration was basally elevated near the Zusanli acupuncture point prior to needle stimulation [9], indicating a pool of AMP was present under baseline conditions. Manual stimulation of the acupuncture needle produced further increases in AMP concentration. In light of these findings, we reasoned that it might be possible to generate additional adenosine and activate A_1_R in the vicinity of acupuncture points by injecting an enzyme that hydrolyzes AMP to adenosine.

We previously found that the transmembrane isoform of prostatic acid phosphatase (PAP) functions as an ectonucleotidase and hydrolyzes extracellular AMP to adenosine in nociceptive dorsal root ganglia neurons [10,11]. PAP is expressed in several other tissues, including skeletal muscle that surrounds the Zusanli acupuncture point, and could be the rate-limiting ectonucleotidase at this location [9,12]. PAP is a very stable enzyme when administered *in vivo*, with an 11.7 d half-life in blood [13]. Likewise, we found that intrathecal injection of a secretory version of human PAP (hPAP) had long-lasting (3 days), A_1_R-dependent antinociceptive effects in pre-clinical models of inflammatory pain and neuropathic pain [10,14]. These long-lasting antinociceptive effects could be transiently blocked with a short-acting A_1_R antagonist, providing strong evidence that hPAP remains in tissue for days [10,15]. In contrast, adenosine has a very short half-life in blood (a few seconds) [16]. hPAP injections thus provide a novel way to generate a short-acting compound over a sustained time period [17].

Here, we sought to determine if the adenosine receptor dependent antinociceptive effects of acupuncture could be exploited and greatly extended by injecting hPAP into an acupuncture point. When combined with a recombinant version of hPAP [18], this approach could be utilized to more effectively treat chronic pain in localized regions of the body without acupuncture needle stimulation.

## Results

**Figure 1:**
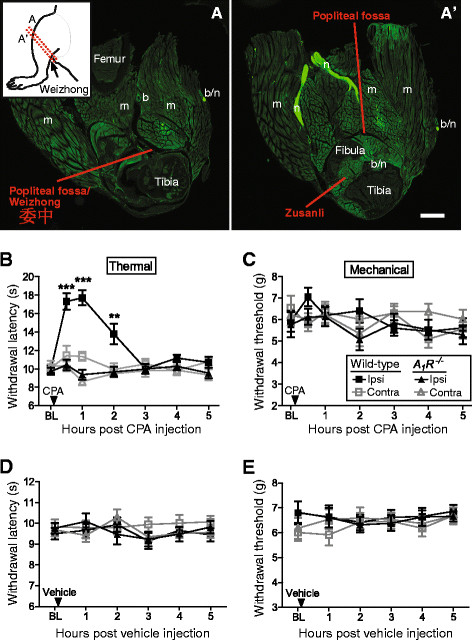
**Injection of an A**_**1**_**R agonist into the Weizhong acupuncture point (located within the popliteal fossa) has short-lasting antinociceptive effects.** (**A-A’**) Cross section of the mouse hind limb cut through knee and popliteal fossa, shows proximity of Weizhong (BL40) and Zusanli (ST36) acupuncture points to each other and to peripheral nerves. Bones, nerves (n), blood vessels (b), muscle (m) and acupoints labeled. Tissue stained with PGP9.5 antibody (1:500) to visualize nerves. (**B**) Noxious thermal and (**C**) mechanical sensitivity in hindpaw following injection of CPA (5 nmol) into the popliteal fossa. (**D**) Noxious thermal and (**E**) mechanical sensitivity following injection of vehicle into the popliteal fossa (0.9% saline with 1% DMSO). (**B-E**) Ipsi = ipsilateral hindpaw. Contra = contralateral hindpaw. Contralateral popliteal fossa was not injected. Wild-type (n = 10) and *A*_*1*_*R*^*−/−*^ (n = 10) male mice in each cohort. Data are plotted as means ± s.e.m. Paired t-tests were used to compare responses at each time point to baseline (BL). *P < 0.05, **P < 0.005, ***P < 0.0005.

The Weizhong acupuncture point, located behind the knee within the popliteal fossa (Figure 1A), is commonly needled by acupuncturists to treat pain [7,19]. Clinicians inject local anesthetics into this same location for regional anesthesia [20,21]. Since A_1_R activation in peripheral tissues, including at the Zusanli acupuncture point (which is anatomically close to Weizhong; Figure 1A’), had brief antinociceptive effects in rodents [9,22-25], we first sought to determine if A_1_R activation at Weizhong also had antinociceptive effects. To test this, we injected a selective A_1_R agonist, N^6^-cyclopentyladenosine (CPA), into the popliteal fossa of wild-type mice and *A*_*1*_*R*^*−/−*^ mice while monitoring noxious thermal and mechanical sensitivity. We found that CPA transiently (2 hr) inhibited noxious thermal sensitivity in the ipsilateral hindpaw of wild-type mice but did not affect thermal sensitivity in the contralateral hindpaw (Figure 1B). In contrast, CPA had no effect in either hindpaw of *A*_*1*_*R*^*-/-*^ mice (Figure 1B). Additionally, CPA did not affect mechanical sensitivity in naïve (non-sensitized) wild-type or *A*_*1*_*R*^*−/−*^ mice (Figure 1C). Lastly, vehicle injection did not affect thermal or mechanical sensitivity (Figure 1D,E). Taken together, these data reveal that activation of A_1_R at the Weizhong acupuncture point can locally inhibit nociception.

**Figure 2:**
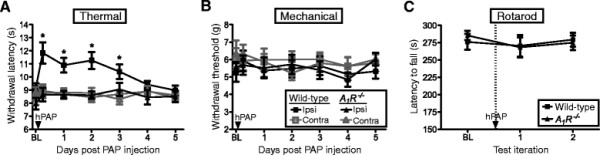
**Peripheral injection of PAP into Weizhong acupuncture point (“PAPupuncture”) has localized and long-lasting A**_**1**_**R-dependent antinociceptive effects.** (**A**) Noxious thermal and (**B**) mechanical sensitivity following injection of hPAP (250 mU) into the popliteal fossa. (**C**) Rotarod tests 24 h before (1 test iteration) and 24 h after (2 test iterations, separated by 40 s) injection of hPAP into the popliteal fossa (250 mU/mouse). No significant differences in rotarod performance between genotypes or treatment. (**A-C**) Ipsi = ipsilateral hindpaw. Contra = contralateral hindpaw. Contralateral popliteal fossa was not injected. Wild-type (n = 10) and *A*_*1*_*R*^*−/−*^ (n = 10) male mice in each cohort. Data are plotted as means ± s.e.m. Paired t-tests were used to compare responses at each time point to baseline (BL). *P < 0.05, **P < 0.005, ***P < 0.0005.

Goldman and colleagues recently found that the concentration of AMP was elevated relative to other nucleotides near the Zusanli acupuncture point [9]. Extracellular AMP is also basally elevated in human muscle [26], the tissue where acupuncture points are located [2]. Given our previous work with PAP—an ectonucleotidase that hydrolyzes AMP to adenosine and activates A_1_R for days following a single spinal injection [10,14,15,17]—we hypothesized it might be possible to enzymatically hydrolyze the pool of AMP to adenosine and inhibit nociception without performing acupuncture. To test this, we injected hPAP protein into the popliteal fossa—a novel approach we call “PAPupuncture”—then monitored noxious thermal and mechanical sensitivity in the hindpaw. Strikingly, injection of hPAP (250 mU) into the popliteal fossa inhibited thermal sensitivity for three days only in the injected leg of wild-type mice (Figure 2A). This localized effect contrasts with spinal injections, which target both legs, and systemic drug injections that target the entire body. Importantly, hPAP did not affect thermal sensitivity in *A*_*1*_*R*^*−/−*^ mice, demonstrating a critical requirement for A_1_R activation (Figure 2A). In addition, hPAP did not affect mechanical sensitivity in naïve mice (Figure 2B). Increased withdrawal latency in the thermal assay was not due to motor impairment, as the same dose of hPAP did not affect motor function in the rotarod test (Figure 2C). These results are the first to reveal that a single, peripheral injection of hPAP locally inhibits nociception for an extended period of time by engaging an A_1_R-dependent mechanism that is common to acupuncture.

**Figure 3:**
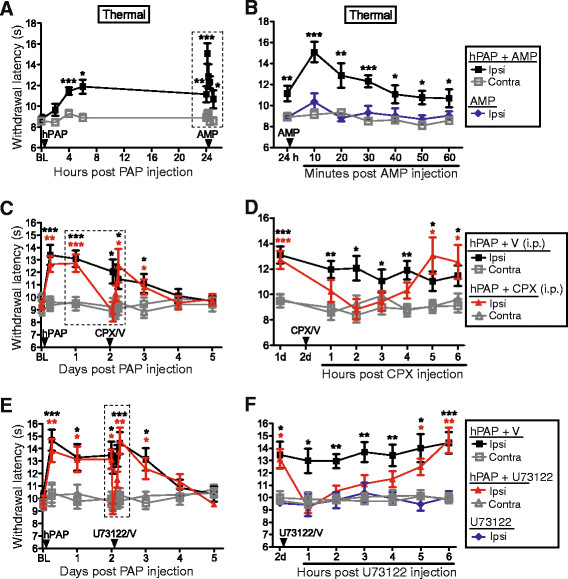
**Local antinociceptive effects of PAP can be transiently boosted by injecting substrate or transiently blocked by interfering with A**_**1**_**R signaling.** (**A**) hPAP (250 mU) followed one day later by substrate (200 nmol AMP) were injected into the popliteal fossa. (**B**) Data from the boxed area in (**A**) along with response following injection of AMP (200 nmol) alone into popliteal fossa. (**C**) hPAP (250 mU) was injected into the popliteal fossa followed two days later by CPX (1 mg/kg, i.p.). (**D**) Data from the boxed area in (**C**). (**E**) hPAP (250 mU) followed two days later by U73122 (5.4 nmol) were injected into the ipsilateral popliteal fossa. (**F**) Data from the boxed area in (**E**) along with response following injection of U73122 (5.4 nmol) alone into popliteal fossa. (**A-F**) Ipsi = ipsilateral hindpaw. Contra = contralateral hindpaw. Contralateral popliteal fossa was not injected. Wild-type (n = 10) and *A*_*1*_*R*^*−/−*^ (n = 10) male mice in each cohort. Data are plotted as means ± s.e.m. Paired t-tests were used to compare responses at each time point to BL. *P < 0.05, **P < 0.005, ***P < 0.0005.

Since hPAP generates adenosine by dephosphorylating AMP, we next sought to determine if the magnitude of the antinociceptive effect could be increased by injecting additional substrate. To test this possibility, we injected wild-type mice with hPAP (250 mU) then injected these same mice with AMP (200 nmol) twenty-four hours later. AMP injection into the popliteal fossa transiently boosted the magnitude of the antinociceptive effect only in mice previously injected with hPAP (Figure 3A,B). These data indicate that the magnitude of the antinociceptive effect was dependent on substrate availability.

To determine if the long-lasting antinociceptive effects of hPAP were due to sustained A_1_R activation, we next injected hPAP (250 mU) into the popliteal fossa of wild-type mice, then 48 h later injected (i.p.) vehicle or a selective A_1_R antagonist, 8-cyclopentyl-1,3-dipropylxanthine (CPX). CPX, but not vehicle, transiently blocked the thermal antinociceptive effect of hPAP for four hours (Figure 3C,D). In contrast, neither vehicle nor systemic CPX affected thermal sensitivity in the contralateral (non-hPAP injected) hindpaw. These data indicate the local antinociceptive effects of hPAP are due to sustained A_1_R activation.

We previously found that the antinociceptive effects of hPAP, when injected intrathecally, were due to A_1_R activation and phospholipase C (PLC)-mediated hydrolysis of phosphatidylinositol 4,5-bisphosphate [14]. To determine if the antinociceptive effects of PAPupuncture were PLC-dependent, we injected hPAP (250 mU) then 48 h later injected vehicle or U73122 (5.4 nmol) into the ipsilateral popliteal fossa. U73122 is commonly used to inhibit PLC-dependent signaling. U73122, but not vehicle, transiently blocked the thermal antinociceptive effect of hPAP for four hours (Figure 3E,F). U73122 alone had no effect on noxious thermal sensitivity (Figure 3F). Thus, hPAP inhibits nociception through a PLC-dependent mechanism when injected peripherally.

**Figure 4:**
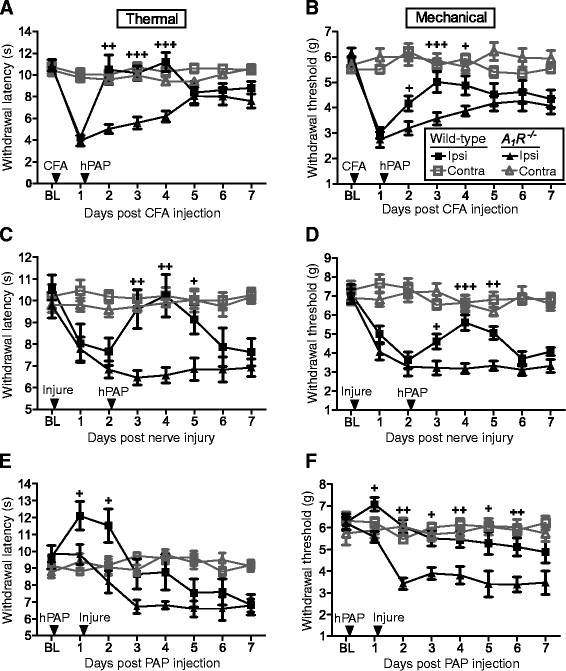
**PAP has long-lasting, localized antinociceptive effects in chronic inflammatory and neuropathic pain models.** (**A, B**) CFA was injected into one hindpaw. One day later hPAP (250 mU) was injected into the ipsilateral popliteal fossa. Inflamed and non-inflamed (contralateral) hindpaws were tested for (**A**) thermal and (**B**) mechanical sensitivity. (**C, D**) The sural and common peroneal branches of the sciatic nerve were ligated and then transected (injure-arrow). Two days later, hPAP (250 mU) was injected into the ipsilateral popliteal fossa. Injured and non-nerve injured (contralateral) hindpaws were tested for (**C**) thermal and (**D**) mechanical sensitivity. (**E, F**) Preemptive study. hPAP (250 mU) was injected into the popliteal fossa. One day later, SNI surgery was performed on the ipsilateral leg. Injured and non-nerve injured (contralateral) hindpaws were tested for (**E**) thermal and (**F**) mechanical sensitivity. (**A-F**) Ipsi = ipsilateral hindpaw. Contra = contralateral hindpaw. Contralateral popliteal fossa was not injected. Wild-type (n = 9-10) and *A*_*1*_*R*^*−/−*^ (n = 10) male mice in each cohort. Data are plotted as means ± s.e.m. T-tests were used to compare responses at each time point between genotypes, same paw comparisons. ^+^*P* < 0.05, ^++^*P* < 0.005, ^+++^*P* < 0.0005.

We next evaluated whether PAPupuncture could block hypersensitivity associated with inflammatory pain and neuropathic pain. To test this, we inflamed one hindpaw with complete Freund’s adjuvant (CFA) then injected hPAP (250 mU) one day later into the ipsilateral popliteal fossa. Strikingly, thermal sensitivity returned to baseline levels and mechanical sensitivity returned almost to baseline for three days in wild-type mice but not *A*_*1*_*R*^*−/−*^ mice (Figure 4A,B). Likewise, in the spared nerve injury (SNI) model of neuropathic pain [27], hPAP (250 mU) inhibited thermal and mechanical hypersensitivity for three days in the ipsilateral (injured) hindpaw of wild-type mice but not *A*_*1*_*R*^*−/−*^ mice (Figure 4C,D). In both chronic pain models, ipsilateral hPAP injections had no effects in the contralateral (non-inflamed/non-injured) hindpaw (Figure 4A-D). Collectively, these data indicate that hPAP has localized, long-lasting, A_1_R-dependent antinociceptive effects in two models of chronic pain when injected peripherally.

To determine if peripheral injection of hPAP could preemptively inhibit pain hypersensitivity, as occurs when hPAP is injected intrathecally [14], we injected hPAP (250 mU) into the popliteal fossa of wild-type and *A*_*1*_*R*^*−/−*^ mice and performed the SNI surgery the following day. hPAP reduced nerve-injury induced thermal hyperalgesia for two days in wild-type but not *A*_*1*_*R*^*−/−*^ mice (Figure 4E). In addition, injection of hPAP prior to nerve injury reduced mechanical allodynia for six days in wild-type mice (Figure 4F), three days longer than when hPAP was injected after nerve injury (Figure 4D). However, given that hPAP did not enduringly reduce hyperalgesia and allodynia for >7 d as hPAP did when injected intrathecally [14], we conclude that hPAP does not have preemptive antinociceptive effects when administered peripherally.

**Figure 5:**
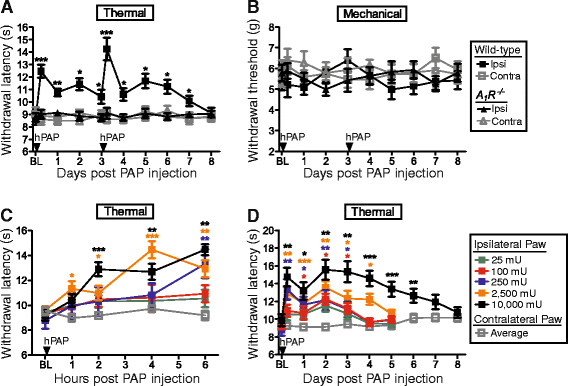
**Larger hPAP doses extend the antinociceptive effects of PAPupuncture.** (**A**) Thermal and (**B**) mechanical sensitivity following sequential injections of hPAP (250 mU/injection) into popliteal fossa. (**C, D**) hPAP dose response. Thermal sensitivity measured in (**C**) hours and (**D**) days post hPAP injection (single injection of the indicated doses). (**A-D**) Ipsi = ipsilateral hindpaw. Contra = contralateral hindpaw. Contralateral popliteal fossa was not injected. Wild-type (n = 10) male mice in each cohort. Data are plotted as means ± s.e.m. Paired t-tests were used to compare responses at each time point to BL. *P < 0.05, **P < 0.005, ***P < 0.0005.

There are several possible reasons why hPAP (250 mU) consistently had antinociceptive effects that lasted three days but not longer: 1) hPAP depleted the local AMP pool, 2) A_1_R desensitized or 3) hPAP protein lost catalytic activity or washed out. To distinguish between these possibilities, we injected wild-type mice and *A*_*1*_*R*^*−/−*^ mice with hPAP (250 mU) then injected these same mice with a second dose (250 mU) three days later. We reasoned that the second hPAP injection should have no antinociceptive effects if the first hPAP injection depleted AMP or desensitized A_1_R. On the contrary, thermal withdrawal latency increased in the ipsilateral hindpaw after the second hPAP injection and remained significantly elevated for four more days in wild-type mice but not *A*_*1*_*R*^*−/−*^ mice (Figure 5A). These sequential hPAP injections did not affect mechanical sensitivity in naïve mice (Figure 5B). However, sequential hPAP injections (250 mU on days 21 and 24 post SNI in the ipsilateral popliteal fossa) had a statistically significant antiallodynic effect that lasted for five days (data not shown). These data reveal that PAPupuncture duration can be extended in naïve and sensitized animals by injecting additional enzyme and that duration is not limited by substrate availability or A_1_R desensitization. Unexpectedly, these results also suggest that the extracellular AMP pool cannot readily be depleted, perhaps reflective of the fact that surrounding muscle generates large quantities of ATP and other nucleotides to power movement.

**Figure 6:**
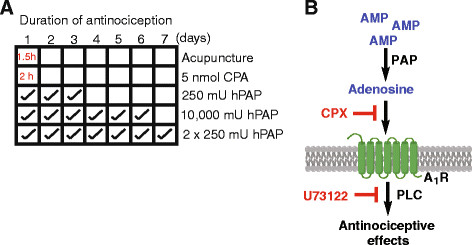
**PAPupuncture duration of action and mechanism.** (**A**) Acupuncture and an adenosine receptor agonist (CPA) have A_1_R-dependent antinociceptive effects that last for 1–2 hr [9]. In contrast, PAPupuncture has antinociceptive effects that last for nearly a week following a single injection or longer following sequential injections. (**B**) PAPupuncture mechanism. Following peripheral injection, PAP locally inhibits nociception via an A_1_R and PLC-dependent pathway. The long-lasting effects of PAP can be transiently blocked with CPX (A_1_R selective antagonist) or U73122 (blocks PLC-dependent signaling), revealing that PAP generates adenosine and activates A_1_R for days without desensitizing receptors.

Since hPAP availability was the limiting factor, we next hypothesized that larger quantities of hPAP might have greater and longer-lasting effects on nociception. Indeed, hPAP dose-dependently inhibited the magnitude and duration of noxious thermal sensitivity (Figure 5C,D), with the highest dose (10,000 mU, single injection into popliteal fossa) lasting for six days—nearly 100x longer than acupuncture (which lasts up to 1.5 hr, Figure 6A) [9]. PAPupuncture antinociception was delayed by 1–6 hr (depending on hPAP dose; Figure 5C), consistent with hPAP working through an enzymatic process and not a pharmacological process (contrast with rapid onset following CPA injection; Figure 1B).

## Discussion

While numerous studies indicate that acupuncture is effective for treating some types of pain, the duration of action is limited [1,6-8]. Acupuncture points are located in muscle in close proximity to peripheral nerves (Figure 1A,A’) [2], an anatomical location that is ideal for intercepting pain signals before they reach the brain. Indeed, Goldman and colleagues found that needle stimulation caused the localized release of nucleotides and adenosine at acupuncture points followed by short-lasting A_1_R-dependent antinociceptive effects [9]. Acupuncture also reduced noxious stimulus-evoked brain activation [9]. While the localized actions of acupuncture are, in principle, ideal for treating pain in specific regions of the body, the short duration of action leaves much room for improvement. Localized actions could also reduce or eliminate side-effects associated with systemic analgesic drug administration.

PAP can rapidly generate adenosine from AMP and is endogenously found in nociceptive neurons and in nociceptive axon terminals [10,11]. However, at the time we began this study, the extent to which PAP or any other ectonucleotidase was directly involved in adenosine-dependent antinociceptive mechanisms in the periphery, including at acupuncture points, was unknown. Dephosphorylation of AMP to adenosine is rate limiting in the periphery and in the brain [9,28], causing extracellular AMP levels to be basally elevated. As is true for any biochemical process, increasing the concentration of a rate-limiting enzyme yields more product (in this case adenosine). In light of this information, we hypothesized that injection of additional hPAP—an enzyme that is endogenously present in muscle tissue surrounding acupuncture points [9,12]—could be used to locally generate adenosine in muscle and near peripheral nerves. Indeed, injection of hPAP into the Weizhong acupuncture point had antinociceptive effects that lasted substantially longer (nearly 100x) than acupuncture (Figure 6A). Our study is the first to show that PAP inhibits nociception via an A_1_R- and PLC-dependent mechanism in the periphery (Figure 6B), highlighting a role for this ectonucleotidase in peripheral pain mechanisms.

Given that a selective A_1_R antagonist blocked the antinociceptive effects of PAPupuncture (CPX, Figure 3), other compounds that block A_1_R, such as theophylline and caffeine (a non-selective A_1_ and A_2_ antagonist), could also reduce PAPupuncture efficacy. This possibility could be particularly relevant if PAPupuncture is advanced into the clinic, as patients should probably eliminate caffeine (and other xanthine-derived alkaloid) intake to maximize analgesic efficacy. Notably, transcutaneous electrical nerve stimulation (TENS) is used clinically to reduce the intensity and unpleasantness of pain in humans, and caffeine (but not placebo) at a dose equivalent to two to three cups of coffee can block these analgesic effects of TENS [29].

Clinicians inject local anesthetics into the popliteal fossa to treat pain following foot and ankle surgery. However, this regional anesthesia procedure requires catheterization to block pain for more than a day [21,30]. Local nerve blocks are administered at many other locations of the body to regionally treat pain. While our work was focused on the popliteal fossa, PAPupuncture could in principle be performed in any body region where acupuncture and nerve blocks are performed and has the potential to reduce pain for a significantly longer period of time. Given that PAP works via an A_1_R-dependent mechanism, PAPupuncture would also bypass side-effects associated with opioid-based analgesics, and hence could provide a novel abuse-resistant way to treat pain. Ultimately, our study reveals that key mechanisms associated with Eastern and Western medicine can be merged and exploited to locally inhibit acute and chronic pain for an extended period of time.

## Methods

### Behavior and injections

All procedures and behavioral experiments involving vertebrate animals were approved by the Institutional Animal Care and Use Committee at the University of North Carolina at Chapel Hill. All behavior experiments were performed with male mice during the light phase, raised under a 12:12 light:dark cycle and fed Prolab RMH 3000 (LabDiet) *ad libitum*. C57BL/6 mice (2–6 months in age) were purchased from Jackson Laboratories, and *A*_*1*_*R*^*−/−*^ mice were backcrossed to C57BL/6 J mice for 12 generations [31]. All mice were acclimated to the experimenter, the room and the experimental apparatus for 3–5 days prior to behavioral testing. Thermal sensitivity was monitored using the Hargreaves method, where the radiant heat source was calibrated to elicit a paw withdrawal reflex of approximately 10 s in naïve mice (cutoff time of 20 s) as previously described [10]. Mechanical sensitivity was measured with semi-flexible tips attached to an electronic Von Frey apparatus (IITC Life Science).

Unless otherwise specified, all compounds were injected in a 50 μL volume in 0.5 mL syringes with a 29 gauge, 0.5 inch needle (BD Biosciences) into the popliteal fossa and diluted in 0.9% saline [32,33]. Mice were anesthetized with isoflurane (Baxter) prior to injection. Human prostatic acid phosphatase (hPAP), N^6^-cyclopentyl adenosine (CPA, 5 nmol in 1% DMSO) and 8-cyclopentyl-1,3-dipropylxanthine (CPX, 1 mg/kg in 0.9% saline with 5% DMSO and 1.25% 1 M NaOH, injected i.p., 100 μL) were from Sigma-Aldrich and prepared as previously described [10]. U73122 (Tocris, 5.4 nmol) was diluted in 50% DMSO and 50% saline [14]. CFA (20 μL, MP Biomedicals) was injected under the glabrous skin to inflame one hindpaw. Spared nerve injury (SNI) was used to model neuropathic pain [27]. Motor function was measured by the rotarod performance test (cutoff time of 300 s). Baseline measurements were collected with 1 test iteration for each mouse. Trials were again performed 24 h following injection of hPAP into the popliteal fossa, with 2 iterations separated by 40 s for each mouse. Statistical significance was calculated using Student’s two-tailed t-test. All error bars are shown as standard error of the mean (± s.e.m.).

### Immunohistochemistry

Mice were anesthetized with pentobarbital (50 mg/kg, i.p.) and perfused intracardially with 4% paraformaldehyde (PFA, 10 mL) in 0.1 M phosphate buffer (PB), pH 7.4. The hindlimbs were removed and post-fixed for 24 hr at 4°C in 50 mL 4% PFA, washed for 6 h at room temperature in 50 mL PBS and decalcified with 50 mL Rapid Cal Immuno (BBC Biochemical) for 3 days at 4°C. The tissue was then cryoprotected in 30% sucrose in PB (50 mL) for 5 days at 4°C before sectioning on the cryostat. Sections of the hindlimb (40 μm) were thaw-mounted onto slides. PGP 9.5 immunostaining was performed as previously described [34].

## Misc

Summary: Acupuncture mechanism can be exploited to provide extremely long-lasting pain relief.

## Competing interests

MJZ is listed as an inventor on a patent application that was licensed to a commercial entity for the purpose of developing PAP as a treatment for pain.

## Author contributions

JKH performed the experiments. JKH and MJZ designed the experiments, interpreted the data and wrote the manuscript. Both authors read and approved the final manuscript.
